# Macroclimate and Topography Interact to Influence the Abundance of Divaricate Plants in New Zealand

**DOI:** 10.3389/fpls.2020.00507

**Published:** 2020-05-19

**Authors:** Christopher H. Lusk, Susan K. Wiser, Daniel C. Laughlin

**Affiliations:** ^1^Environmental Research Institute, University of Waikato, Hamilton, New Zealand; ^2^Manaaki Whenua – Landcare Research, Lincoln, New Zealand; ^3^Department of Botany, University of Wyoming, Laramie, WY, United States

**Keywords:** browsing, divaricating plants, drought, frost, landform, plant structural defenses, soil nitrogen, soil phosphorus

## Abstract

The abundance of the divaricate growth form in New Zealand has been interpreted as either (a) the response of an isolated flora to cool, dry, Plio-Pleistocene climates; or (b) a defense against large browsing birds (moa) that were hunted to extinction shortly after human arrival during the last millennium. We used patterns of divaricate plant abundance across present-day landscapes to test a novel synthetic hypothesis: that the divaricate form is of most value to plants on fertile soils that attract herbivores, on sites where climatic constraints prevent plants from quickly growing out of the browse zone. This hypothesis predicts that divaricate species should be most abundant on terraces (landforms that are both fertile and frost-prone) in regions that are cold and dry, and should be scarce across all topographic positions in the warmest (largely frost-free) regions. To address our hypothesis, we first tested the influence of topography on frost regimes and nutrient levels by measuring temperatures and soil total C, N, and P at four standard topographic positions at five localities differing widely in macroclimate. We then extracted a dataset of 236 surveys comprising 9,877 relevé plots from the New Zealand National Vegetation Survey databank. We calculated the proportion of arborescent species with a divaricate growth form and the proportion of total arborescentcover contributed by divaricates on each plot; we then fitted linear mixed-effect models predicting these response variables as functions of topographic position and climate. The number of frosts recorded averaged <1 yr^–1^ at the warmest of the five sites studied, to >60 yr^–1^ on all topographic positions at the coldest site. Terraces were subject to more frequent and harder frosts than any other topographic position. Topography had no significant influence on total N or C:N, but total P was higher on terraces and in gullies than on faces or ridges. Frost-free period was the dominant influence on both species representation and cover of divaricate plants throughout the country. The effect of topography was also significant, but weaker. The effect of frost-free period was stronger on sites with water deficits than on sites where precipitation exceeded evapotranspiration. Divaricates made their largest contributions on terraces in cold, dry regions; as predicted, they were scarce on all topographic positions on sites with frost-free periods >300 days. Our hypothesis was generally supported, although the effect of topography on divaricate abundance was not as strong as some previous studies led us to expect. Divaricates made their largest contributions to arborescent species richness and cover on sites where climatic restrictions on growth coincide with relatively high nutrient availability. The contemporary distribution of the divaricate form across New Zealand landscapes thus appears to be reasonably well explained by the hypothesized interaction of climate and fertility-mediated browsing, although experiments may provide more conclusive tests of this hypothesis.

## Introduction

More than 120 years after the first treatment of New Zealand’s divaricate plants in the scientific literature (e.g., Diels, 1897), scientists continue to debate the causes of the local abundance of this distinctive growth form (Bond et al., 2004; Lusk et al., 2016; Wood and Wilmshurst, 2017). In New Zealand, the term “divaricate” has been applied to 50–60 woody species from at least 17 different families, with small leaves, long internodes and wide branching angles, often resulting in an interlacing crown (Greenwood and Atkinson, 1977; Kelly, 1994). Divaricate branches have higher tensile strength than those of non-divaricate congeners (Pollock et al., 2007) or of adults in the case of heteroblastic species that are only divaricates as juveniles (Bond et al., 2004). Diels (1897) suggested the divaricate form conferred resistance to harsh climates, and arose in response to the Plio-Pleistocene onset of frosty, droughty environments. McGlone and Webb (1981) further developed this hypothesis, arguing that the divaricate form represents the evolutionary response of an isolated and essentially subtropical flora to the climatic fluctuations of the Plio-Pleistocene period, and that it may now enable plants to cope with the unpredictable occurrence of frost, drought and wind in modern New Zealand’s oceanic climates.

Empirical tests of the climatic hypothesis have been largely inconclusive (Kelly and Ogle, 1990; Darrow et al., 2002; Howell et al., 2002; Lusk and Clearwater, 2015). However, recent modeling confirms an association with frosty and droughty habitats: divaricate species contribute most to arborescent assemblages in the eastern South Island, on sites with cold winters and where evapotranspiration exceeds precipitation; they are also well represented in the central North Island, where the frost-free period is <4 months on many sites (Lusk et al., 2016). Similarly, congeneric contrasts show that divaricate plants are consistently associated with environments that are on average frostier than those of their closest broadleaved relatives (Garrity and Lusk, 2017).

In contrast, Greenwood and Atkinson (1977) contended that the divaricate form arose as a defense against unique local browsing pressures. New Zealand is unique among sizeable landmasses in lacking native browsing mammals; in prehistoric New Zealand the only terrestrial browsers were birds, the largest of which were nine species of flightless moa (Dinorthiformes) that were hunted to extinction shortly after human arrival during the last millennium (Anderson, 2003; Perry et al., 2014). Although contemporary New Zealand ecosystems lack exact functional analogs of moa (Forsyth et al., 2010; Lee et al., 2010), introduced ungulates tend to avoid most (but not all) divaricate plants (Forsyth et al., 2002; Pollock et al., 2007), despite the high leaf nutrient levels of some species (Lee and Johnson, 1984). Divaricates are not the only element of New Zealand’s woody flora that is unattractive to ungulates: most taxa with sclerophyllous foliage are also avoided (Forsyth et al., 2002; Tanentzap et al., 2011). The moa-browsing hypothesis received support from an experimental study of browsing by caged extant ratites from other regions (emu and ostrich), which were unable to feed effectively on the divaricate juvenile forms of two heteroblastic New Zealand trees, but rapidly consumed (non-divaricate) adult foliage (Bond et al., 2004). A similar “wire plant” syndrome in semi-arid southwestern Madagascar has also been attributed to coevolution with extinct avian browsers (Bond and Silander, 2007).

Although debate about divaricate plants has often been polarized, it has also been suggested that a synergy of climatic and browsing pressures might ultimately be implicated (Wardle, 1985; Cooper et al., 1993). Here we use patterns of divaricate plant abundance across present-day landscapes to address a novel synthetic hypothesis: that the divaricate form (like other putative physical defenses against browsers) is of most value to plants on fertile sites that attract herbivores, and where climatic constraints prevent plants from quickly growing out of the browse zone (Lusk et al., 2016). Greenwood and Atkinson (1977) stated that large concentrations of divaricate plants often occur on the fertile alluvial soils of terraces, and argued this reflected selection for defense against the high browsing pressure resulting from the attraction of herbivores to nutrient-rich environments (e.g., Grubb, 1992; Kanowski et al., 2001). Subsequent studies have confirmed that terraces (and gullies) are generally more nutrient-rich than nearby ridges or slope faces (Richardson et al., 2008; Jager et al., 2015). McGlone and Webb (1981), however, noted that terraces are also generally the most frost-prone parts of landscapes (Laughlin and Kalma, 1987). A recent modeling study failed to detect any effect of topographic position on the representation of divaricate species in arborescent assemblages, which was predicted best by macroclimatic variables (Lusk et al., 2016). Here we use a larger dataset and a new modeling approach to test the effects of macroclimate and topographic position on divaricate abundance. Our synthetic hypothesis predicts that divaricates (1) should increase in abundance with increasing frostiness and/or water deficit, and (2) should show a bias toward terraces (fertile, frost-prone) except in frost-free areas where they should be scarce on all topographic positions. In contrast, the browsing hypothesis predicts a nationwide association with both gullies and terraces (generally the most fertile parts of landscapes) but not necessarily any effect of macroclimate; and we interpret the climate hypothesis as predicting divaricates to be common anywhere subject to short frost-free periods and/or to water deficits.

## Materials and Methods

### Study Area

The three main islands of New Zealand span c. 12.5° of latitude, from 34.5° to 47° S. The South Island is mountainous; the main axial ranges running the length of the island including many peaks >2000 m. Quaternary glaciation has been influential in shaping South Island landforms, and extensive outwash plains occur to the east of the ranges. In the North Island the ranges are lower, and only three young andesitic cones exceed 2000 m. Rugged hill country makes up much of the North Island, but an extensive rhyolitic plateau occupies the center, and several of the major rivers have formed sizeable alluvial plains and terrace systems. The smaller Stewart Island is mostly undulating, with low ranges not exceeding 1000 m elevation.

Although New Zealand climates are broadly described as oceanic temperate, they encompass a wide range of temperature regimes and annual precipitation (Garnier, 1958; NIWA, 2001). Mean annual temperatures range from <5°C at high elevations in the south to c. 16°C in the far north. Frosts are rare on western coasts of the North Island but occur throughout most of the year in intermontane basins in the eastern South Island and in the central North Island. Rainfall patterns on the South Island are dominated by the strong orographic effect of the axial ranges (Southern Alps): the moisture-laden prevailing westerly winds bring >2000 mm annually to most sites west of the main divide (and often >4000 mm at high elevations), whereas the eastern lowlands in the lee of the Southern Alps mostly receive <1000 mm. The west-to-east precipitation gradient is less marked in the North Island, because of the lower and more complex relief. Some alluvial lowland areas in the east and south-west of the North Island receive <1000 mm; at the other extreme, >2000 mm falls on the major volcanoes and on most ranges above 1000 m elevation. Many North Island sites are subject to a slight rainfall minimum in summer, but precipitation is evenly distributed throughout the year over most of the South Island. Stewart Island is wet (>1500 mm) and cool throughout, with a strong maritime influence buffering daily and seasonal temperate variation.

Before human arrival in the late 13th century, most of New Zealand was covered by closed-canopy forest, with tussock grasslands above treeline, and extensive wetlands in some lowland areas (Wardle, 1991). Lowland plains and terraces of the North and South Islands have been almost entirely converted to intensive agriculture, with only small scattered forest remnants surviving. The only exception to this generalization is the wet western coast of the South Island, where extensive lowland forests remain on poorly drained terraces. Lowland hill country has been largely deforested in the east of both main islands, but extensive native forest cover remains on these landforms in the western North Island and the southern South Island. The main axial ranges are still largely covered in primary forest, although human set fires have caused an expansion of tussock grasslands. Extensive seral shrublands occur on both main islands. In contrast to the North and South Islands, Stewart Island remains largely forested.

Although moa are thought to have been extinct for at least 500 years, most New Zealand forests are now inhabited by a range of browsing ungulates introduced during the 19th century (King, 2005). Selective browsing by ungulates has strongly influenced the understorey composition of many New Zealand forests (Wardle et al., 2001), and many divaricate species are among the plants that are avoided or not selected by ungulates (Forsyth et al., 2002; Pollock et al., 2007; Lusk, 2014).

### Plot Data

Compositional data from vegetation plots were obtained from the New Zealand National Vegetation Survey (NVS) databank (Wiser et al., 2001). We chose 236 native forest and shrubland surveys to maximize the range of temperature and site water balance in our dataset. These comprised 10,495 relevé plots on which abundance of each vascular plant species was recorded within height tiers using a cover-abundance scale of six classes: <1, 1–5, 6–25, 26–50, 51–75, ≥76% (Hurst and Allen, 2007). In order to exclude any sites where significant invasion of exotic trees such as *Pinus* spp. had occurred, we omitted any plots with ≥10% cover of exoticspecies.

To minimize biases caused by human-set fires, we excluded any plots with ≥25% cover of *Leptospermum scoparium* or *Kunzea* spp. (Myrtaceae), the most common woody colonizers of burnt sites throughout New Zealand. Our rationale for this was that human settlement during the last millennium has vastly increased fire frequencies (Ogden et al., 1998), and that there is a strong topographic bias in the distribution of fires, ridges being burnt far more often than gullies (Perry et al., 2010). As divaricate plants are often common in the early stages of secondary succession (McGlone and Webb, 1981), inclusion of fire-induced seral vegetation might therefore have misled us about the topographic distribution of this group of plants. Although both *L. scoparium* and *Kunzea* spp. do sometimes occur in undisturbed forest on poor soils, they are normally abundant in only early-successional stands (e.g., Allen, 1988; Mark et al., 1989).

We computed the proportion of divaricate species in the arborescent assemblage present on each plot (Supplementary Data Sheet 1). This variable represents the degree of dominance of the divaricate form on each plot, and has been shown to correlate more strongly with climate than actual divaricate species richness (Lusk et al., 2016). We also estimated the proportion of arborescent cover contributed by divaricate plants at each site, after converting cover scores of each species within each height tier to the midpoint of the corresponding cover-abundance class. The summed cover of the *n* divaricate species on a given plot was estimated as:

$$C_{d}=\sum_{i=1}^{n}\max\left\{T_{1},T_{2},\ldots \,T_{N}\right\}$$

where *T*_1_ is the cover score of the *i*th species in tier 1, *T*_2_ is the score in tier 2, etc. As most divaricate species are shrubs or small trees, their maximum cover scores were most often found in the understorey or subcanopy. The same procedure was used to estimate the summed cover of all indigenous arborescent species on the plot (*C*_*t*_). The proportion of cover contributed by divaricate species was thus computed as *C_*d/*_C_*t*_*.

Climatic and lithological variables were obtained from GIS surfaces. We used the mean annual frost-free period, sourced from the Global Agro-ecological Zones portal (Fischer et al., 2008) as our macroclimatic indicator of frost regimes. We used the mean annual water deficit [AWD; available through the Land Resource Information Systems (LRIS) portal (Landcare Research, 2011)] as our indicator of site water balance. AWD is based on a water balance model derived from spatially explicit predictions of mean daily temperature, mean daily solar radiation, and mean rainfall. These predictions are themselves derived from primary climate data and physiographic variables such as elevation and aspect (Leathwick et al., 2002). Potential evaporation is calculated following Priestley and Taylor (1972). AWD layer is then computed as the sum of any monthly deficits between rainfall and potential evaporation. Topographic positions of plots were recorded when the compositional data were collected as one of four classes (ridge, face, terrace, and gully) or using a 9-point topographic scale (Dalrymple et al., 1968), which we condensed to the four classes.

After obtaining the climatic data, we edited the dataset to minimize the impact of climatic and topographic bias in the survival of indigenous forest throughout the country. Although abundant forest has survived on all topographic positions in high-rainfall districts, cold dry sites are highly underrepresented in the NVS data because they have been largely deforested since human arrival. Most notably, our dataset included no terrace plots on sites where annual water deficits coincided with frost-free periods <130 days. We therefore curtailed our analysis to sites with ≥100 frost-free days, in order to limit the influence of questionable extrapolations that might distort our findings. This step reduced the number of plots analyzed to 9,601.

### Influence of Topography on Soil Nutrients and Frost Regimes

We quantified the influence of topography on soil nutrients and frost regimes at five localities differing widely in temperature and site water balance (Table 1). The environmental data obtained from these five localities was intended to inform our interpretation of the distribution patterns of divaricate plants as indicated by our compositional database. 89.5% of the plots in our compositional database fell within the range of annual water deficit represented by our five chosen localities, and 98.8% of plots fell within the corresponding range of frost-free period.

**TABLE 1:** Summary of nested ANOVA testing effects of locality and topographic position on frost regimes, across five localities differing widely in macroclimate.

**Effect**	**SS**	**df**	**MS**	***F***	***p***
**(A) No. of air frosts**					
Intercept	44917	1	44917	926.8	<0.00001
Site	53056	4	13264	273.7	<0.00001
Topography(Site)	1713	15	114.2	2.356	0.012
Error	2374	49	48.47		
**(B) Lowest recorded temperature**					
Intercept	109.4	1	109.4	77.78	<0.00001
Site	321.6	4	80.41	57.18	<0.00001
Topography(Site)	49.17	15	3.278	2.331	0.013
Error	68.90	49	1.406		

*At each locality, temperatures were measured from spring 2017 to spring 2018, at three replicate ridges, terraces and gullies, and up to six replicate faces.*

**FIGURE 1:**
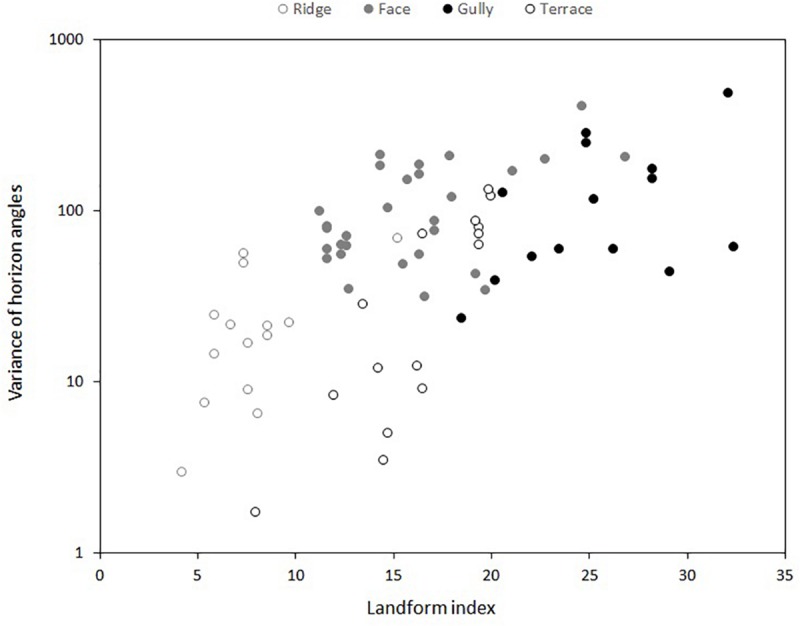
Relationship between landform index (McNab, 1993) and four subjectively-determined topographic positions at five different localities. Landform index is the mean of the angle to the horizon in eight equidistant compass directions at each sampling point. *Y*-axis shows the variance of these eight horizon angles at each sampling point.

Four standard topographic positions were recognized: ridge, face, terrace, gully (Whitehouse et al., 1990; Richardson et al., 2008). At each of the five localities, frost regimes and soil nutrients were measured at three replicate ridge, terrace and gully sites, and at up to six faces—the greater replication of faces resulted from the initial selection of both north- and south-facing slopes. Although these replicates were chosen subjectively, we verified our choices by measuring the landform index proposed by McNab (1993). This involves measuring the angle to the horizon in eight equidistant compass directions—the average angle is typically lowest on ridges, and highest in gullies (McNab, 1993; Richardson et al., 2008). We found the landform index distinguished well between our ridge, terrace and gully sites, but was less successful in separating faces from terraces or gullies, reflecting the variety of landscapes encompassed by our five localities (Figure 1). Nevertheless, it proved useful to consider the variance of the eight horizon angles at each site as well as the mean, as the variance tended to be lower on terraces than on faces (Figure 1).

At each replicate site, we installed an iButton Thermachron DS1912 temperature logger (Maxim Integrated, San Jose, CA, United States) to measure air temperatures beneath a canopy gap resulting from the recent death of a single overstorey tree. We regarded temperatures beneath canopy gaps as more informative than those beneath closed forest canopies, which buffer maximum and minimum air temperatures (e.g., Caramori et al., 1996; Lusk and Laughlin, 2017). Radiation shields, custom-made from stacked plastic plates (Tarara and Hoheisel, 2007), were used to protect temperature loggers against radiative heating and chilling. Temperature loggers were positioned at 1m above the forest floor and programed to record relative humidity and air temperatures (to the nearest 0.5°C) every 4 h. The memory of the DS1912 can store up to 341 days’ measurements at this frequency. Loggers were installed at the five localities between September and November 2017, meaning the measurement period ran from spring 2017 to spring 2018 at all sites.

Samples of the uppermost 15 cm of topsoil were taken at the same replicated topographic positions at each locality. At each replicate site, soil was extracted at five haphazardly chosen points, and homogenized for determination of soil total carbon (C), nitrogen (N), and phosphorus (P). At each locality, there were thus three to six replicate measurements of total C, N, and P on ridges, terraces and in gullies, and up to six replicate measurements on faces. Data from 26 sites distributed across diverse New Zealand landscapes (Parfitt et al., 2005) indicated that soil C:N and total P were strongly correlated respectively with leaf N (*r* = −0.67) and P (*r* = 0.82) of the widely-distributed tree *Weinmannia racemosa*, suggesting these soil variables are reasonable indicators of N and P availability to plants. Total C and N were determined by near infra-red spectroscopy, calibrated by Dumas combustion. Total P was determined by nitric/hydrochloric acid digestion followed by inductively coupled plasma - optical emission spectrophotometry.

### Analysis

Temperature records downloaded from the iButtons were used to compute two variables summarizing frost regimes. We computed the average number of air frosts and the average minimum temperatures experienced over the 341-day measurement period, on each topographic position at each locality. We then used nested ANOVA to test effects of locality and topographic position on frost regimes (Table 1). Nested ANOVA were also used to test effects of locality and topography on soil total N, C:N ratio and total P (Table 2).

**TABLE 2:** Summary of nested ANOVA testing effects of locality and topographic position on topsoil chemistry, across five localities differing widely in macroclimate.

**Effect**	**SS**	**df**	**MS**	***F***	***p***
**(A) (log) total N (%)**					
Intercept	13.29	1	13.292	604.1	<0.00001
Site	1.810	4	0.4750	21.62	<0.00001
Topography(Site)	0.4729	15	0.03153	1.435	0.166
Error	1.164	53	0.02197		
**(B) C:N ratio**					
Intercept	16958	1	16958	1310	<0.00001
Site	762.7	4	190.7	14.73	<0.00001
Topography(Site)	315.7	15	21.05	1.626	0.098
Error	686.1	53	12.95		
**(C) (log) total P (ppm)**					
Intercept	425.2	1	425.2	24014	<0.00001
Site	0.9844	4	0.2461	13.90	<0.00001
Topography(Site)	0.9740	15	0.0649	3.670	0.00023
Error	0.9385	53	0.0177		

*At each locality, samples were obtained from the uppermost 15 cm of soil at three to six replicate ridges, terraces and gullies and up to six replicate faces.*

We used the nlme R package (Pinheiro et al., 2011) to fit linear mixed effect models to determine which factors explain divaricate species’ representation in arborescent assemblages and their proportional contribution to total cover of arborescent species. These were modeled as functions of the interactions among topography, frost-free period, and water deficit occurrence. A square-root transformation was applied to divaricate species’ representation and proportion divaricate cover in order to improve the distribution of the proportional response variable. The vast majority of observations were proportions <0.4, so this transformation upweights small positive values. Water deficit occurrence was coded as a binary variable: 1 if a deficit was present, and 0 if not. Each model included an exponential correlation structure using latitude and longitude of plots to account for spatial autocorrelation in the dataset because plots were clustered within individual surveys across the country. Lithological classes of soil parent materials were initially included, but later dropped as this variable explained very little variation.

## Results

### Frost Regimes and Soil Nutrients

**FIGURE 2:**
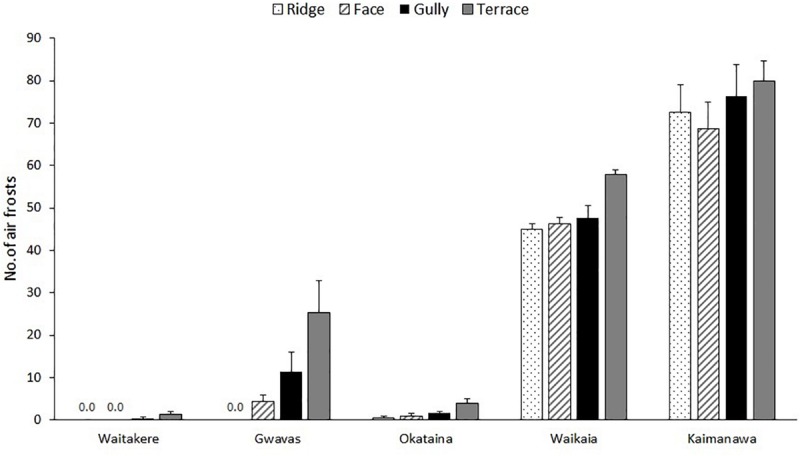
Effect of topography on frequencies of air frosts at 1m aboveground beneath single-tree canopy gaps, at five localities differing widely in macroclimate, over a 340-day period between spring 2017 and spring 2018. Graph shows means (±1 se) of measurements at three to six replicate ridges, terraces and gullies and up to six replicate faces.

Frost regimes differed widely between the five localities (Figure 2), and there was also a weaker but significant effect of topographic position on both air frost frequencies and lowest recorded temperatures (Tables 1, 3). Terraces were generally the frostiest topographic position, and ridges the least affected by frost at most localities (Figure 2). Air frosts were frequent (>40) on all topographic positions at the two coldest localities (Kaimanawa and Waikaia), but rare or absent on all topographic positions at the warmest locality (Waitakere). The effect of topography on lowest recorded temperatures was greatest at the mild and dry Gwavas site, where air temperatures on terraces fell to 2.8°C below the minima recorded on ridges; and least pronounced at the cold and wet Kaimanawa site, where only 1.3°C separated the minima recorded on terraces and ridges (Table 3).

**TABLE 3:** Effect of topography on minimum temperatures and soil nitrogen, at five localities differing widely in macroclimate.

**Locality**	**Parent material**	**Macroclimate**			**Topographic position**	**Lowest recorded temperature (°C) (spring** **2017-spring 2018)**	**N content of top 15 cm mineral soil**	
		**MAT (°C)**	**Frost-free period (days)**	**Annual water deficit (mm)**			**Total N (%)**	**C:N**
**Waitakere**	Andesitic lavas and conglomerates	14	335	13	Ridge	3.3 ± 0.7	0.50 ± 0.05	17.4 ± 1.3
36.8865° S					Face	3.3 ± 0.7	0.47 ± 0.05	15.3 ± 1.3
174.5225° E					Terrace	0.8 ± 0.8	0.50 ± 0.10	16.3 ± 2.3
					Gully	0.7 ± 0.3	0.47 ± 0.04	14.5 ± 0.8
**Gwavas**	Loess/gravels	11.9	232	81	Ridge	0.8 ± 0.2	0.61 ± 0.06	11.3 ± 0.1
39.7703° S					Face	−1.4 ± 0.4	0.69 ± 0.09	11.6 ± 0.3
176.4716° E					Terrace	−2.0 ± 0.3	0.54 ± 0.06	11.0 ± 0.2
					Gully	−1.7 ± 0.3	0.74 ± 0.12	11.0 ± 0.5
**Okataina**	Basaltic and rhyolitic tephras	11.8	195	0	Ridge	0.0 ± 0.5	0.34 ± 0.03	12.8 ± 0.8
38.0841° S					Face	0.3 ± 0.2	0.26 ± 0.04	13.6 ± 0.5
176.4228° E					Terrace	−1.3 ± 0.5	0.47 ± 0.06	15.9 ± 0.9
					Gully	−0.5 ± 0.3	0.30 ± 0.03	14.0 ± 1.5
**Waikaia**	Greywacke	9.8	115	54	Ridge	−2.8 ± 0.2	0.15 ± 0.02	26.5 ± 0.9
45.5554° S					Face	−1.9 ± 1.2	0.16 ± 0.01	24.3 ± 1.7
169.0168° E					Terrace	−3.5 ± 0.0	0.29 ± 0.05	16.7 ± 1.3
					Gully	−3.2 ± 0.3	0.28 ± 0.02	18.3 ± 1.9
**Kaimanawa**	Andesitic and rhyolitic tephras	8.5	99	0	Ridge	−4.8 ± 0.8	0.45 ± 0.13	21.0 ± 2.8
39.2361° S					Face	−3.9 ± 0.4	0.26 ± 0.02	18.3 ± 2.6
175.7801° E					Terrace	−5.2 ± 0.3	0.43 ± 0.18	18.1 ± 1.3
					Gully	−4.5 ± 0.5	0.31 ± 0.04	16.6 ± 2.0

*Table shows mean of 3–6 replicates, ±1 sd. Frost frequencies and soil total *P* data are shown in Figures 2, 4. Climatic variables and soil parent material were obtained from the Land Resource Information Systems (LRIS) portal (Landcare Research, 2011) and the Global Agro-ecological Zones portal (Fischer et al., 2008).*

**FIGURE 3:**
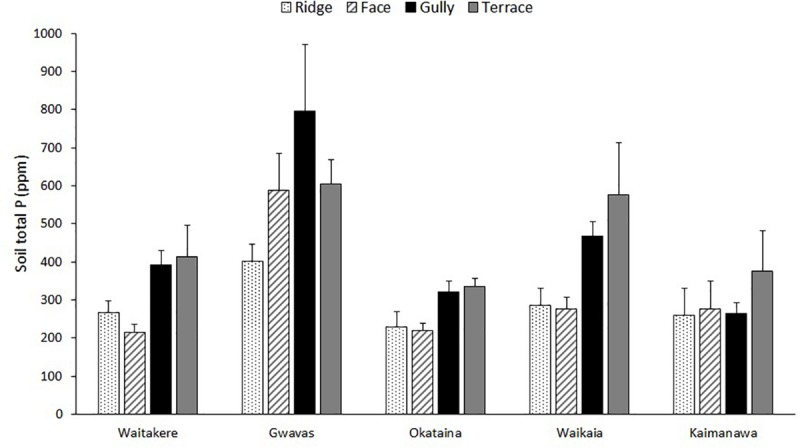
Effect of topography on total P of uppermost 15 cm of mineral soil, at five localities differing widely in macroclimate. Graph shows means (±1 se) of measurements at three to six replicate ridges, terraces and gullies and up to six replicate faces.

The five localities differed widely in soil total N, C:N ratio, and total P; but the effect of topography was less consistent (Tables 2, 3 and Figure 3). Terraces and gullies were generally richer in total P than faces or ridges, but topography had less influence on soil C:N, and no influence on total N (Tables 2, 3 and Figure 3).

### Representation of Divaricate Species in Arborescent Assemblages

**FIGURE 4:**
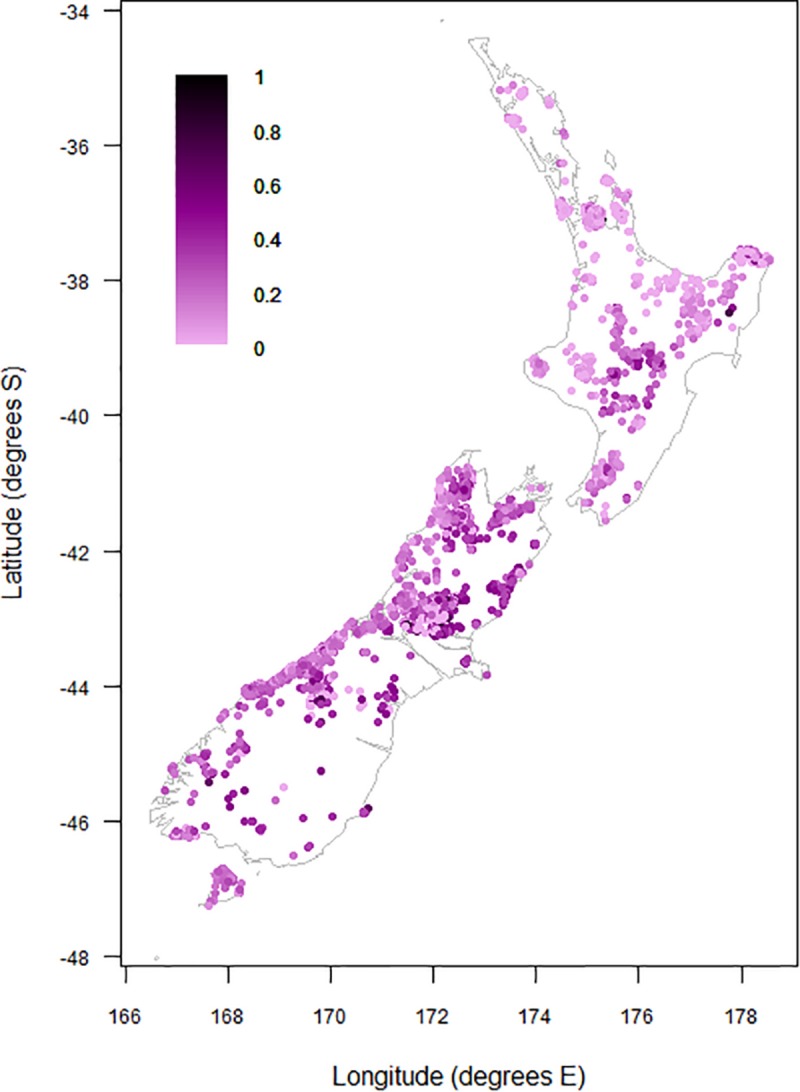
Proportional contribution of divaricate species to arborescent assemblages on 9,601 relevé forest plots on the main islands of New Zealand.

Divaricate representation was generally highest in the eastern South Island, where divaricate species often comprised >50% of arborescent assemblages (Figure 4). Figures exceeding 40% were recorded on some inland sites in the middle North Island. Divaricates generally contributed least to arborescent assemblages on coastal sites in the northern and western North Island, where figures exceeding 20% were uncommon (Figure 4).

**FIGURE 5:**
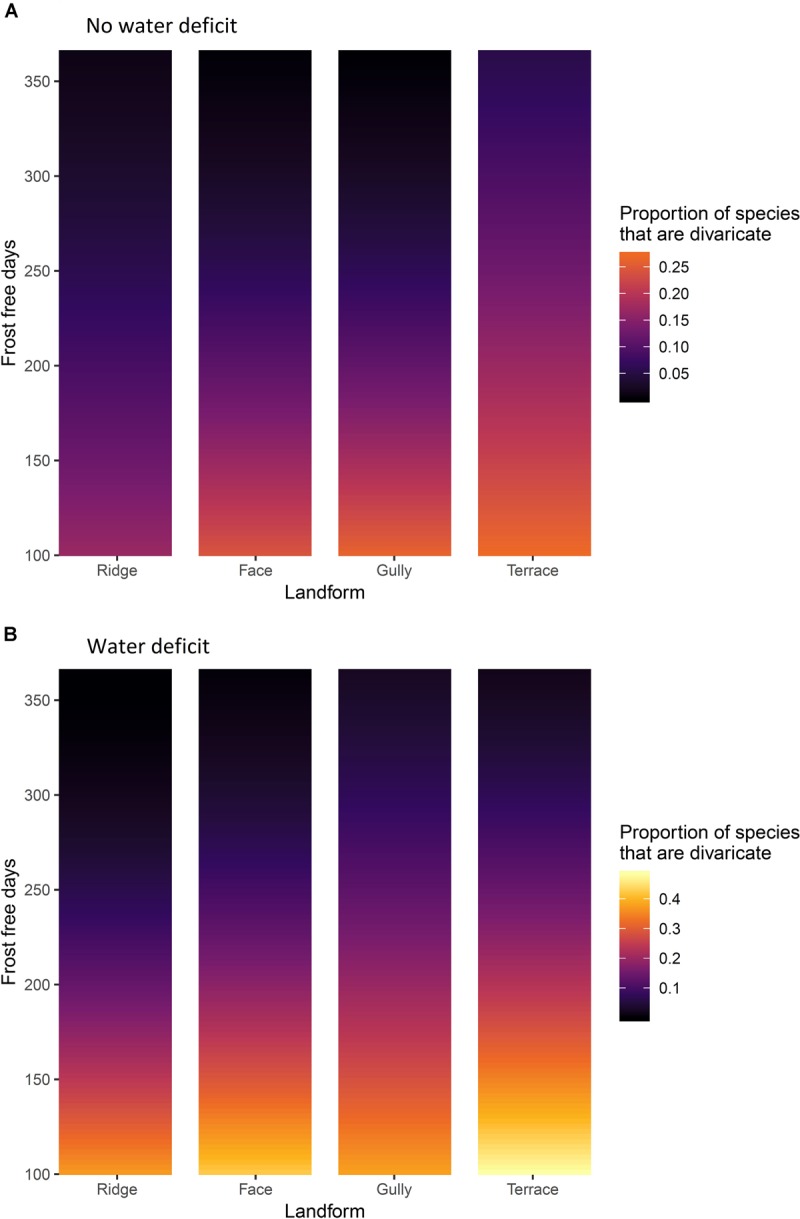
**(A)** Sites without water deficit, i.e. precipitation exceeds evapotranspiration in all months of the year. **(B)** sites with an annual water deficit, i.e. where evapotranspiration exceeds precipitation during some months. The effects of frost-free period and topographic position on representation of divaricate species in arborescent assemblages on 9,601 relevé forest plots in New Zealand. Yellow = high representation of divaricate species, black = low representation.

Climate and topographic position explained 25.1% of variation in the proportion of divaricate species in woody assemblages. All three main fixed effects were significant (Table 4). Frost-free period was the dominant effect, with divaricate representation consistently increasing with decreasing frost-free period on all topographic positions, irrespective of whether or not the site is subject to a water deficit (Figure 5). There was also a weaker but highly significant effect of topography, divaricates being best represented on terraces. Divaricate species representation was generally higher on sites with water deficit than on sites without deficit, this effect again occurring across all topographic positions.

**TABLE 4:** ANOVA of effects of topography and macroclimate on proportion of divaricate species in arborescent assemblages across 9,601 plots, by Satterthwaite’s method.

**Effect**	**DF**	***F*-value**	**Pr(>*F*)**
(Intercept)	1	33440	<0.0001
Topography	3	100.5	<0.0001
Frost-free period	1	2362	<0.0001
Water deficit	1	103.0	<0.0001
Topography × frost-free period	3	13.00	<0.0001
Topography × water deficit	3	9.090	<0.0001
Frost-free period × water deficit	1	89.49	<0.0001
Topography × frost-free period × water deficit	3	6.510	0.0002

All interactions were also significant, though weaker than the main effects (Table 4). The most influential interaction was between frost-free period and water deficit, the effect of water deficit being minimal on warm sites and most evident on the frostiest sites (Figure 5). The effect of topographic position on divaricate representation tended to become more accentuated with decreasing frost-free period (Figure 5), though this interaction was weaker than that between frost-free period and water deficit (Table 4). Water deficit also exerted a weak modulating influence on the effect of topography (Table 4), which was stronger on average on sites where evapotranspiration exceeded rainfall (Figure 5). There was a weak but significant interaction between all three main effects (Table 4), presumably driven mostly by a divergence between gullies and terraces–whereas divaricate representation was most responsive to frost-free period on terraces in areas with a water deficit, it was the assemblages of gullies that responded most strongly on wet sites (Figure 5).

### Cover of Divaricate Species

**FIGURE 6:**
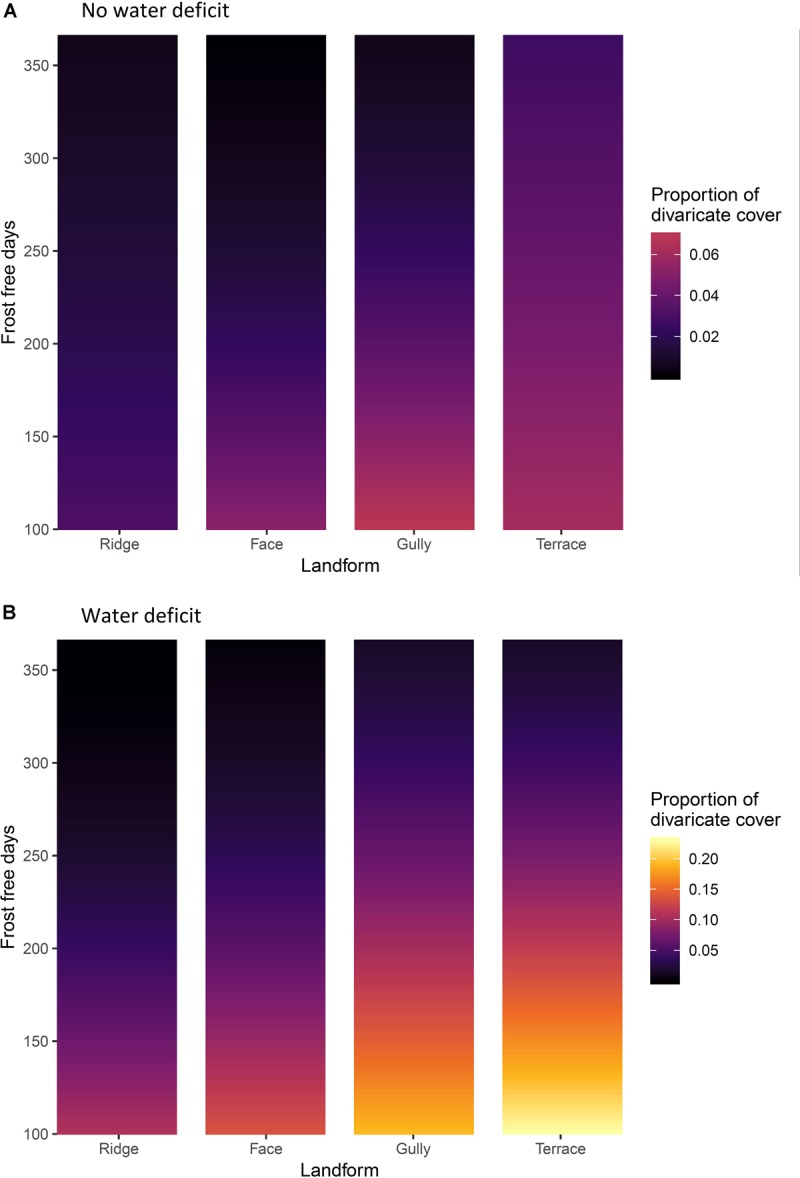
**(A)** Sites without water deficit, i.e. precipitation exceeds evapotranspiration in all months of the year. **(B)** sites with an annual water deficit, i.e. where evapotranspiration exceeds precipitation during some months. The effects of frost-free period and topographic position on divaricate species’ cover on 9,601 relevé forest plots in New Zealand. Yellow = high cover of divaricate species, black = low cover.

Climate and topography explained 12.4% of variation in the proportion of total cover contributed by divaricates. Frost-free period was again the dominant influence on proportional cover of divaricates, though water deficit had more influence on cover than it did on species representation (Table 5). The effect of topography was also significant (Table 5), divaricates making a larger average contribution on terraces and in gullies than on faces or ridges (Figure 6).

**TABLE 5:** ANOVA of effects of topography and macroclimate on of proportion of cover contributed by divaricates across 9,601 plots, by Satterthwaite’s method.

**Effect**	**DF**	***F*-value**	**Pr(>*F*)**
(Intercept)	1	13095	<0.0001
Topography	3	96.55	<0.0001
Frost-free period	1	692.8	<0.0001
Water deficit	1	202.5	<0.0001
Topography × frost-free period	3	9.077	<0.0001
Topography × water deficit	3	9.947	<0.0001
Frost-free period × water deficit	1	97.55	<0.0001
Topography × frost-free period × water deficit	3	3.860	0.009

The dominant interaction was again that of frost-free period with water deficit (Table 5), the effect of water deficit being minimal on warm sites and most evident on the frostiest sites (Figure 6). The effect of topographic position on divaricate cover tended to become more accentuated with decreasing frost-free period and was also more evident on sites with water deficits (Figure 6); although both these effects were weaker than the interaction of frost-free period with water deficit (Table 5). The interaction between all three main effects fell short of statistical significance (Table 5).

## Discussion

The data were mostly consistent with our hypothesis that the divaricate form is of most value to plants on fertile soils, on sites where climatic constraints prevent plants from quickly growing out of the browse zone. Divaricates were most prominent on terraces in cold, dry areas; and scarce on all topographic positions in the warmest parts of the country (Figures 5, 6). Support for our hypothesis was clearest on sites with water deficits, where it was on terraces (the most frost-prone topographic position) that divaricate abundance increased most noticeably as frost-free period decreased (Figures 5B, 6B). However, on wet sites both species representation and cover of divaricates on terraces were relatively unresponsive to frost regimes, and it was in gullies that their importance increased most as frost-free period decreased (Figures 5A, 6A).

As climate limits species-specific maximum heights of woody plants (Ryan and Yoder, 1997; Wang et al., 2012), the harshest climates could confine some trees and shrubs to the browse zone throughout their entire lives. The cold, dry climates prevailing over much of New Zealand during glacial periods are likely to have produced such an effect, resulting in strong selection for anti-browsing defenses over much of the landscape. However, there appears to have been widespread survival of small pockets of forest on sites where climate was buffered by topography and/or proximity to the sea (McGlone et al., 2016); broadleaved species more attractive to browsers might have been largely confined to these sites where local climates gave them some chance of escaping the browse zone.

Our temperature measurements confirmed the expected influence of topography on frost regimes (Figure 2). Terraces were generally the frostiest parts of landscapes, reflecting ponding of cold air draining from surrounding higher ground at night (Laughlin and Kalma, 1990; Kalma et al., 2012). On the warmest sites, terraces were the only topographic position where air frosts occurred with any frequency; whereas at the coldest localities, frosts occurred frequently on all topographic positions, though a weak effect of topography was still apparent at Waikaia (Figure 2). In the only directly comparable New Zealand study that we are aware of, Richardson et al. (2013) reported only a weak effect of topographic position on winter air temperatures beneath small canopy openings in a central North Island forest.

The expected influence of topography on soil fertility was only partly confirmed (Figure 3). Topography had a significant effect on only total P, and this was dwarfed by overall differences between the five localities sampled (Table 2). At least two previous studies have also reported a stronger influence of topography on soil total P than on total N or C:N in New Zealand forest landscapes (Richardson et al., 2008; Jager et al., 2015). However, strong topographic gradients of N availability have sometimes been reported elsewhere (e.g., Garten et al., 1994; Tateno and Takeda, 2003), and it is not clear what factors are responsible for this difference. Topographic gradients in total P at our sites were weaker than those reported in previous New Zealand studies (Richardson et al., 2008; Jager et al., 2015), possibly reflecting the prevalence of more gentle relief at most of our chosen localities. The high landform index values (McNab, 1993) of the gullies studied by Richardson et al. (2008) suggest they worked in a more steeply-dissected landscape conducive to downslope transport of nutrients. Furthermore, the most steeply-dissected of our five landscapes (Kaimanawa) is subject to regular interruption of pedogenesis by tephra deposition from the active central North Island volcanoes (Lowe, 2010), a factor that might also slow the development of strong topographic gradients of P.

Our results appear to parallel the reported distribution of the more typical anti-browsing defense of spinescence in Africa, where plant growth is restricted more by aridity than by frost. Milton (1991) reported that spinescent plants are commonest on “run-on” landforms (terraces and gullies) and on nutrient-rich parent materials in low-rainfall areas of southern Africa. Similarly, Cowling and Campbell (1983) found that spinescence in the eastern Cape region is more common on relatively fertile soils than in fynbos shrublands on very poor soils nearby. These appear to be other examples of physical defenses against browsing being most strongly favored where high nutrient availability coincides with climatic restrictions on plant growth rates. More recently, a continent-wide study showed the incidence of spinescence across Africa to be controlled primarily by aridity, with spinescent species generally contributing <10% of rainforest arborescent assemblages, rising to >25% in semi-arid savannah environments (Charles-Dominique et al., 2016). Some spinescent species of African savannas also have cage-like architectures not unlike those of New Zealand divaricate plants (Charles-Dominique et al., 2017).

Although the results are more consistent with the synthetic hypothesis than with explanations based solely on either climate or browsing, direct physiological advantages associated with the divaricate form may also contribute to the patterns reported here (McGlone and Webb, 1981; Lusk et al., 2016). In addition to leaving juvenile plants more exposed to ground-dwelling herbivores by curtailing the growing season, frost and drought also pose well-known direct physiological challenges to plants. Small leaves such as those of divaricate plants are less vulnerable to chilling on clear nights than broad leaves (Lusk et al., 2018). Small leaves are also less susceptible to overheating when exposed to high radiation loads during rainless periods (Yates et al., 2010; Leigh et al., 2017), conditions that are most likely to occur on sites with annual water deficits.

The overall effect of topography on divaricate abundance was not as strong as some previous studies led us to expect. Exceptionally large concentrations of divaricate plants have been recorded on terraces in mudstone-dominated inland areas of the middle North Island (Clarkson and Clarkson, 1994; Lusk, 2014). However, our dataset included few plots on such sites (reflecting the scarcity of forest remnants on these prime agricultural sites), and similarly, strong effects of topography on divaricate abundance were not apparent in other parts of New Zealand. Soils developed from mudstone are among the most fertile in the country (New Zealand and Soil Bureau, 1968); as vertebrate herbivore biomass is reported to be positively correlated with soil fertility (Peres, 1997; Kanowski et al., 2001), it is possible that anti-browsing defenses are especially advantageous on terraces derived from mudstone and other nutrient-rich parent materials.

This study advances on previous work (Lusk et al., 2016) by showing that topography influences the distribution of divaricate plants in New Zealand. Our analysis of a large nationwide dataset showed that divaricates make their largest contributions to arborescent species richness and cover on sites where climatic restrictions on growth coincide with high nutrient availability. The contemporary distribution of the divaricate form across New Zealand landscapes thus appears to be reasonably well explained by the hypothesized interaction of climate and fertility-mediated browsing (Lusk et al., 2016), although experiments may provide more conclusive tests of this hypothesis.

## Data Availability Statement

All datasets generated for this study are included in the article/Supplementary Material.

## Author Contributions

CL and SW designed the study. SW compiled the vegetation plot data from diverse sources in the NVS Databank. DL and CL analyzed the data. CL led the writing of the manuscript with contributions from all authors. All authors approved the final version of the manuscript.

## Conflict of Interest

The authors declare that the research was conducted in the absence of any commercial or financial relationships that could be construed as a potential conflict of interest.

## Supplementary Material

DATA SHEET S1 Locations of 10,495 plots obtained from New Zealand National Vegetation Survey, used to examine patterns of divaricate species representation in arborescent assemblages. Topographic positions and climate data are also provided.
